# The European Prescribing Exam: assessing whether European medical students can prescribe rationally and safely

**DOI:** 10.1007/s00228-022-03301-6

**Published:** 2022-03-03

**Authors:** Erik M. Donker, David J. Brinkman, Milan C. Richir, Paraskevi Papaioannidou, Robert Likic, Emilio J. Sanz, Thierry Christiaens, João N. Costa, Fabrizio De Ponti, Ylva Böttiger, Cornelis Kramers, Michiel A. van Agtmael, Jelle Tichelaar, On behalf of the Erasmus+ consortium EuroPE+ and the EACPT Education Working Group, Erik Donker, David Brinkman, Jelle Tichelaar, Milan Richir, Paraskevi Papaioannidou, Robert Likic, Emilio Sanz, Thierry Christiaens, João Costa, Fabrizio De Ponti, Ylva Böttiger, Cornelis Kramers, Michiel Agtmael

**Affiliations:** 1 Section Pharmacotherapy, Department of Internal Medicine, Amsterdam UMC, Location VUmc, De Boelelaan 1117, 1081 HV Amsterdam, The Netherlands; 2 Research and Expertise Centre in Pharmacotherapy Education (RECIPE), Amsterdam, The Netherlands; 3 1st Department of Pharmacology, School of Medicine, Faculty of Health Sciences, Aristotle University of Thessaloniki, Thessaloniki, Greece; 4 Unit of Clinical Pharmacology, Department of Internal Medicine, University Hospital Centre Zagreb and University of Zagreb School of Medicine, Zagreb, Croatia; 5 School of Health Science, Universidad de La Laguna, San Cristobal de La Laguna, Tenerife, Spain; 6 Hospital Universitario de Canarias, La Laguna, Tenerife, Spain; 7 Department of Clinical Pharmacology, Ghent University, Ghent, Belgium; 8 Laboratory of Clinical Pharmacology and Therapeutics, Faculty of Medicine, University of Lisbon, Lisbon, Portugal; 9 Pharmacology Unit, Department of Medical and Surgical Sciences, Alma Mater Studiorum, University of Bologna, Bologna, Italy; 10 Department of Medical and Health Sciences, Linköping University, Linköping, Sweden; 11 Department of Pharmacology-Toxicology, Radboud University Medical Center, Nijmegen, The Netherlands

Many doctors take on prescribing responsibilities shortly after they graduate [1, 2], but final-year medical students not only feel insecure about prescribing, but also lack adequate knowledge and skills to prescribe rationally and safely [3, 4]. To address this public health concern, the European Association for Clinical Pharmacology and Therapeutics (EACPT) recommended that education in clinical pharmacology and therapeutics (CP&T) in Europe should be modernized and harmonized [5]. The first step towards harmonization was taken in 2018 when CP&T experts reached consensus on the key learning outcomes for CP&T education in Europe [6]. The next step was to assess these outcomes in a uniform examination during undergraduate medical training [7–9]. The Prescribing Safety Assessment (United Kingdom) and the Dutch National Pharmacotherapy Assessment (The Netherlands) are currently the only national CP&T examinations [10–13]. Implementing these examinations in other European countries is difficult because of related costs and differences in available drugs and guidelines. Therefore, in 2019, together with nine European universities, the EACPT, and the World Health Organization Europe, we started a 3-year Erasmus + -project (2019–1-NL01-KA203-060,492) to develop, test and implement an online examination on safe prescribing for medical schools in Europe: “The European Prescribing Exam” (EuroPE^+^, https://www.prescribingeducation.eu/). The aim of The European Prescribing Exam is to ensure that medical students in Europe graduate with prescribing competencies for safe and effective clinical practice.

During the first stage of the project, we established that EuroPE^+^ should focus not only on safe prescribing (e.g. contraindications, interactions) but also on broader aspects of CP&T (e.g. deprescribing, communication, personalized medicine). We identified 43 main learning objectives and 299 attainment targets, based on previous European studies of CP&T education and the Dutch National Pharmacotherapy Assessment [6, 14, 15]. The attainment targets concern eight drug groups that junior doctors should be confident about prescribing because these drugs are commonly prescribed or are a major cause of adverse events [16]. Doctors should know the indications for these essential medicines and have the specific knowledge and skills necessary for their safe prescribing. These medicines were chosen based on a Delphi study to determine the medicines that junior doctors in Europe should be able to prescribe safely and effectively [17]. All supplementary files can be found on our website.

During the second stage of the project, we established the assessment blueprint and the digital examination environment. Students will be assessed on all levels of competence according to Miller’s pyramid [18], which is embedded in the blueprint (Fig. 1). The examination includes not only knowledge questions but also skills questions, such as our newly developed case-based question which require students to choose a drug, its route of administration and dosage. All questions are corrected automatically, directly after completion of the approximately 120-min examination. Since the examination focuses on essential knowledge and skills for clinical practice, and assuring patient safety, the pass score is high.

**Fig. 1:**
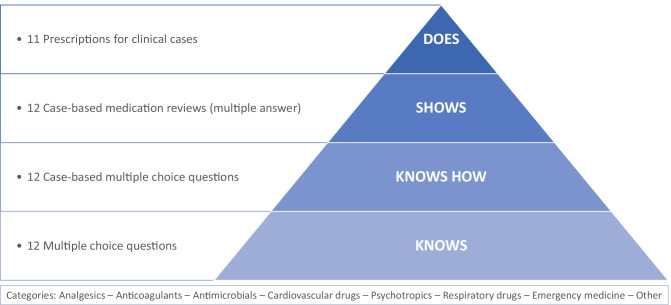
Overview of the European Prescribing Exam

During the third stage of the project, we created a database with peer-reviewed questions submitted by the participating universities. In the future, together with the EACPT Education Working Group, the quality of the questions will be established by an Expert and an Assessment board. The former, consisting of clinical pharmacologists, will develop and review questions, and the latter, consisting of members from different European countries, will oversee that items entered into the question bank are fit for purpose.

During the last year of the grant, we will test the first 200 questions by examining them in two pilot tests on final-year medical students of the participating universities. The European Prescribing Exam is expected to be available for other universities in Europe from September 2022, and most importantly, free of charge. Interested in participating? Please contact the corresponding author.

## Declarations

### Disclaimer

Erasmus + consortium EuroPE^+^

All the listed authors form the consortium.
